# A Web-Based Screening Instrument for Depression and Anxiety Disorders in Primary Care

**DOI:** 10.2196/jmir.5.3.e23

**Published:** 2003-09-29

**Authors:** Peter Farvolden, Carolina McBride, R Michael Bagby, Paula Ravitz

**Affiliations:** ^1^Centre for Addiction and Mental HealthClinical Research DepartmentToronto ONCanada; ^2^Centre for Addiction and Mental HealthToronto ONCanada

**Keywords:** depression, anxiety disorders, assessment of health care needs, screening, web-based services, treatment, primary care, diagnosis, mental health

## Abstract

**Background:**

Major depressive disorder (MDD) and anxiety disorders are common and result in considerable suffering and economic loss. People suffering from major depressive disorder and/or anxiety disorders are commonly encountered in the primary care setting. Unfortunately, most people with these disorders remain either untreated or inadequately treated; current data suggest that general practitioners fail to diagnose up to half of cases of major depressive disorder or anxiety. There is a need for screening tools that will help physicians and other professionals in primary care recognize and adequately treat major depressive disorder and anxiety disorders. While the currently-available self-report screening instruments have been demonstrated to be reliable and valid, there remain considerable barriers to their widespread use in primary care.

**Objective:**

The purpose of the present study is to report preliminary validation data for a freely-available, brief, Web-based, self-report screener for major depressive disorder and anxiety disorders.

**Methods:**

The Web-Based Depression and Anxiety Test (WB-DAT) was administered to 193 subjects who presented for assessment and/or treatment in ongoing research projects being conducted at the Mood and Anxiety Program and Clinical Research Department at the Centre for Addiction and Mental Health in Toronto, Ontario, Canada. Subjects completed the Web-based screening instrument and were subsequently interviewed with the Structured Clinical Interview for the Diagnostic and Statistical Manual of Mental Disorders, fourth edition (DSM-IV) Axis I Disorders (SCID-I/P). The diagnostic data from the screening instrument were then compared with the data from the individual's SCID-I/P interview. Diagnostic concordance between SCID-I/P diagnoses and the Web-Based Depression and Anxiety Test were assessed using Cohen's kappa, sensitivity, specificity, positive predictive value, negative predictive value, and efficiency.

**Results:**

Agreement ranged from acceptable to good (0.57-0.70) for major depressive disorder, panic disorder with and without agoraphobia (PD+/-AG), social phobia/social anxiety disorder, obsessive compulsive disorder (OCD), generalized anxiety disorder (GAD), and post traumatic stress disorder (PTSD). With the exception of generalized anxiety disorder, the sensitivity (0.71-0.95) and specificity (0.87-0.97) for the major diagnostic categories assessed by the Web-Based Depression and Anxiety Test were good. The sensitivity for generalized anxiety disorder was somewhat lower (0.63) but acceptable. Positive predictive values were good (0.60-0.75) for major depressive disorder, obsessive compulsive disorder, generalized anxiety disorder, and post traumatic stress disorder, and acceptable for panic disorder with and without agoraphobia and for social phobia/social anxiety disorder.

**Conclusions:**

These preliminary data suggest that the Web-Based Depression and Anxiety Test is reliable for identifying patients with and without major depressive disorder and the anxiety disorders of panic disorder with and without agoraphobia, social phobia/social anxiety disorder, obsessive compulsive disorder, and post traumatic stress disorder. Further research is required in a larger sample in primary care.

## Introduction

### Major Depressive Disorder, the Anxiety Disorders, and Their Prevalence

Major depressive disorder (MDD) and the anxiety disorders are common and result in significant suffering, lost opportunity, and economic loss. With a prevalence rate of approximately 5% worldwide, MDD is the most common mood disorder [1]. Estimates of lifetime risk for MDD have been reported as 12% for males and 20% for females [2,3]. The average age of onset of MDD is in the third and fourth decade of life. The average length of an untreated major depressive episode is from 6 to 24 months [1]. MDD is often a chronic illness that consists of several major depressive episodes, with the risk of recurrence increasing with each successive episode [4]. Depression profoundly affects quality of life and untreated or inadequately-treated depression is a major public health problem. MDD has become one of the leading causes of morbidity according to the World Health Organization (1997). MDD is projected to become the leading cause of disability and the second-leading contributor to the global burden of disease by the year 2020 [5].

The Diagnostic and Statistical Manual of Mental Disorders, fourth edition (DSM-IV) recognizes a number of distinct anxiety disorders, including specific phobias, social phobia/social anxiety disorder (SP), panic disorder (PD) with and without agoraphobia (PD+/-AG), AG without a history of panic, generalized anxiety disorder (GAD), obsessive-compulsive disorder (OCD), and post-traumatic stress disorder (PTSD). Anxiety disorders are among the most-prevalent psychiatric illnesses. According to the National Comorbidity Survey the lifetime prevalence for all categories of anxiety disorders in the United States is 24.9% [3]. Although anxiety disorders often have their onset in childhood or early adolescence, those afflicted typically do not seek treatment until well into adulthood. Adults with anxiety disorders are at risk for secondary psychiatric comorbidity; significant occupational, educational, and social impairment; and increased need for medical treatment, resulting in enormous economic costs to society [6- 9]. By 2020, PD, OCD, and PTSD will be second only to MDD, and ahead of schizophrenia and alcohol use as a cause of disability world wide [5].

Anxiety disorders have high rates of comorbidity with other psychiatric disorders including other anxiety disorders, MDD, and substance abuse/dependence. Anxiety disorders often occur with MDD. For example, MDD occurs in up to 60% of people with anxiety disorders [10]. Comorbid anxiety and depression is associated with more severe symptoms, impairment, subjective distress, and persistent course of illness than either depression or anxiety alone [11].

### Assessment and Treatment of Major Depressive Disorder and the Anxiety Disorders

In North America, primary care/family medicine practitioners are the primary providers of first-line treatment for MDD and anxiety disorders [12]. People suffering from MDD and anxiety disorders are commonly encountered in the primary care setting, with a prevalence ranging from 5% to 50% [13- 15]. Approximately 50% of people suffering from MDD seek help from their primary care physician [16]. Unfortunately, a large proportion of people who suffer with MDD or an anxiety disorder in the community remain either untreated or inadequately treated [17,18]. Only one half of those with MDD and one third of those with anxiety disorders seek treatment for their illness [16]. More often, depressed and anxious people consult with their primary care physicians for other physical complaints, resulting in increased use of health care services [19,20].

Current data suggest that general practitioners fail to diagnose up to half of cases of depression or anxiety [14]. This situation is unfortunate on at least two counts. First, because it is becoming increasingly clear that people who are adequately treated earlier in their illness have a better prognosis [21]. Second, because once depression and anxiety are accurately recognized, most people with MDD or an anxiety disorder can successfully be managed in primary care using a variety of medications or psychotherapy. For example, cognitive behavioral therapy (CBT) is an effective treatment for both depression and anxiety disorders, and interpersonal psychotherapy (IPT) and cognitive behavioral analysis system of psychotherapy (CBASP) are effective treatments for MDD [13]. However, limited access to evidence-based psychotherapy outside of specialized clinics and research settings often renders pharmacotherapy the most practical first-line treatment option in primary care [22].

There are barriers to better assessment and treatment of MDD and the anxiety disorders in primary care, including a lack of recognition and adequate treatment in primary care, such as a lack of brief, sensitive, easy-to-administer, and easy-to-interpret self-report psychiatric-screening instruments. Without adequate detection and an accurate diagnosis, there cannot be adequate treatment. Establishing an accurate primary diagnosis is important in guiding the specific method and course of treatment [23]. Current evidence suggests that compared with usual care, feedback of depression screening results to providers generally increases recognition of depressive illness in adults [24].

In psychiatry, structured diagnostic interviews are the standard for diagnostic accuracy and are widely employed in research settings. Structured interviews such as the Structured Clinical Interview for DSM-IV Axis I Disorders (Version 2.0/Patient Form) (SCID-I/P) [25] and the Mini-International Neuropsychiatric Interview (MINI) [26] are designed to collect comprehensive data to establish precise diagnoses in the context of research studies. Such interviews take considerable time and must be administered and scored by an expert. As a result, such detailed interviews have not been widely adopted in clinical practice outside of the context of research.

In response to increasing demands for diagnostic precision and accountability in nonresearch clinical settings, there are now reliable and valid screening instruments available for use in primary care including the Primary Care Evaluation of Mental Disorders (PRIME-MD) [27], Symptom-Driven Diagnostic System (SDDS) [28], and MINI-Screen [26]. In general, these instruments are 1-page or 2-page, paper-and-pencil, screening instruments intended to be completed by patients, then hand-scored and interpreted by a health care professional.

While the currently-available self-report screening instruments have been demonstrated to be reliable and valid, there remain considerable barriers to their widespread use in primary care. First, many of the available instruments are narrow in their scope of assessment. For example, there are a large number of 1-page screening instruments designed to assess for the symptoms of MDD, PD, PD+/-AG, social anxiety disorder/social phobia, OCD, GAD, or PTSD. However, given the high rates of comorbidity among these disorders, instruments that assess for only 1 of them are of dubious utility. A major problem with all of the better and broadly-focused DSM-IV screening tools is that they are not freely available. In addition, these instruments all require laborious scoring and interpretation by a health care professional. Given these serious barriers to ease of use, they are unlikely to be widely adopted in primary care no matter how good they are.

### Internet Screening for Major Depressive Disorder and the Anxiety Disorders

The Internet provides an excellent medium for providing patients and health care professionals in primary care access to a brief, algorithm-scored, easily-interpretable self-report screening test for MDD and the anxiety disorders. There are a large number of self-report screeners for anxiety and depression available on the Internet. Unfortunately, they are all subject to the same limitations as the currently-available paper-and-pencil tests insofar as they are all either too limited in scope, not easily scored or interpreted, and/or not freely available. None provide both a broad screen of MDD and the anxiety disorders and few provide any kind of print function that might facilitate a discussion of symptoms with a health care professional in primary care.

Van Mierlo Communications Consulting Inc has recently designed a screening test for MDD and the anxiety disorders that is freely available on the Internet. The screener is currently available as The Depression Test at The Depression Center (http://www.depressioncenter.net/depressiontest) [29], and slightly reconfigured (with questions regarding the anxiety disorders appearing first) as The Anxiety Test at The Panic Center (http://www.paniccenter.net/anxietytest) [30].

This test, the Web-Based Depression and Anxiety Test (WB-DAT) was designed to be a brief, freely-available, Web-based, self-report screening tool for MDD and the anxiety disorders compatible with the DSM-IV and The International Classification of Diseases and Related Health Problems, tenth revision (ICD-10) diagnostic systems [31]. As a screening tool for primary care the instrument was designed to be highly sensitive (ie, to detect a high proportion of patients with a disorder) and reasonably specific (ie, screen out patients without disorders).

Based on their responses to 11 broad preliminary questions based on DSM-IV criteria central to the diagnoses of MDD and each of the anxiety disorders, users are presented with several additional questions for each disorder based on DSM-IV criteria. The result is an algorithm-generated personalized "final report," which summarizes the individual's responses relating to the major diagnostic categories. The final report was designed to be printed and shared with a health care professional.

The WB-DAT was designed to provide a summary of standard diagnostic information in order to initiate and encourage a discussion of specific anxiety and depression symptoms between patients and health care professionals. As a result, there are few diagnostic algorithms to limit the number of diagnoses a health care professional might query. Thus, for example, if a patient meets screening criteria for MDD, GAD, and OCD, the screener summary (final report) will report symptoms of MDD, GAD, and OCD, leaving the diagnostic decision regarding the primary diagnosis and focus of treatment to the health care professional.

In deciding what disorders to screen for in primary care, developers of the test were guided by the diagnostic criteria described in DSM-IV and ICD-10. As a result, the WB-DAT includes screening modules for MDD, PD+/-AG, AG without a history of panic, OCD, social phobia/social anxiety disorder, GAD, PTSD, and acute stress disorder (ASD). The focus of the WB-DAT is on current, rather than past (or lifetime), symptoms and distress/impairment.

Although the WB-DAT has considerable face validity, it is important that the instrument's operating characteristics be evaluated by determining the agreement between the WB-DAT screener diagnoses and diagnoses as made by SCID-I/P. Thus, the purpose of the present study is to report on the operating characteristics of the WB-DAT as compared with gold-standard diagnoses obtained by the SCID-I/P. The WB-DAT was also designed to include additional screening modules for agoraphobia without a history of panic, acute stress disorder, specific phobia, and a number of subsyndromal symptom profiles (for example, symptoms of agoraphobia without significant distress or impairment, dysthymia, and simple phobias) that may aid health care professionals in primary care to reach diagnostic conclusions. However, due to the relatively-small sample size in this study we report here only data for the major diagnostic categories (ie, MDD, PD+/-AG, OCD, SP, GAD, and PTSD).

## Methods

### Participants

The WB-DAT was administered to 193 subjects. All subjects were 18 years of age or older. The sample consisted of 79 (40.9%) men and 114 (59.1%) women. On average, subjects were 40.92 (SD = 12.61) years of age. Subjects with dementia, mental retardation, or serious medical illnesses were excluded.

### Procedure

Subjects were recruited from individuals who presented for assessment and/or treatment in ongoing research projects being conducted at the Mood and Anxiety Program and Clinical Research Department at the Centre for Addiction and Mental Health (CAMH) in Toronto, Ontario, Canada. Projects included 2 ongoing studies of the treatment of MDD, and a study of DSM-IV symptoms and personality in social and problem gamblers. In addition to the standard assessments conducted in the study, interested subjects were asked to consent to participate in the validation study of the WB-DAT.

Subjects completed the WB-DAT using a pseudonym and were subsequently interviewed with the SCID-I/P. The diagnostic data from the WB-DAT were then compared with the data from the individual's SCID-I/P interview. The SCID-I/P was administered by MA-level and PhD-level psychology graduate students who had received formal standardized training, including observing expert-conducted interviews and being observed conducting interviews. Such training has been reported to produce high diagnostic agreement for the DSM-IV Axis I disorders [25]. This study was approved by the Research Ethics Board at the Centre for Addiction and Mental Health, in accordance with applicable regulations, and informed consent was provided.

### Statistical Analyses

Diagnostic concordance with the SCID-I/P was assessed for each Axis-I disorder assessed by the WB-DAT using Cohen's kappa, sensitivity, specificity, positive predictive value, negative predictive value, and efficiency [32- 34]. Cohen's kappa is a correlation of agreement that includes a correction for chance agreement. Sensitivity is the proportion of subjects with a diagnosis by SCID-I/P who receive a positive WB-DAT result (true positives). Specificity, in contrast with sensitivity, is the proportion of subjects without the diagnosis by SCID-I/P who also have a negative WB-DAT result (true negatives). Positive predictive value is the probability of receiving a SCID-I/P diagnosis when restricted to those cases that meet criteria according to the WB-DAT. Negative predictive value is the probability of not receiving a SCID-I/P diagnosis when restricted to all cases that do not receive a WB-DAT diagnosis. Efficiency is a measure of the overall accuracy of the WB-DAT—the number of cases correctly classified by the WB-DAT divided by the sample size.

## Results

Subjects received an average of 0.99 (SD = 1.45) diagnoses according to the WB-DAT and 0.79 (SD = 1.17) diagnoses according to the SCID-I/P. However, only 79/193 (40.9%) of the sample met WB-DAT criteria for 1 or more disorders, and only 78/193 (40.4%) met SCID-I/P criteria for 1 or more disorders. The base rates for both acute stress disorder and AG without a history of panic were too low to permit evaluation of the performance of the WB-DAT for these disorders. The prevalence rates for MDD, any anxiety disorder, and any disorder according to the WB-DAT and the SCID-I/P for the sample are shown in Table 1.

**Table 1:** Prevalence of disorders according to the Web-Based Depression and Anxiety Test and SCID-I/P (n = 193)

	**Web-Based Depression and Anxiety Test**	**SCID-I/P**
Major depressive disorder	51 (26.4%)	48 (24.9%)
Any anxiety disorder	66 (34.2%)	61 (31.6%)
Any disorder	79 (40.9%)	78 (40.4%)

**Table 2:** Operating characteristics of the Web-Based Depression and Anxiety Test compared with SCID-I/P Diagnosis as the gold standard (n = 193)

	**Number** **Meeting** **SCID-I/P** **Criteria**	**Cohen's Kappa**	**Sensitivity**	**Specificity**	**Positive** **Predictive** **Value**	**Negative** **Predictive** **Value**	**Efficiency**
Major depressive disorder	48	0.68	0.79	0.89	0.75	0.93	0.89
Panic disorder +/-agoraphobia	16	0.57	0.75	0.94	0.52	0.98	0.93
Social phobia/social anxiety disorder	19	0.59	0.74	0.94	0.56	0.96	0.93
Obsessive-compulsive disorder	14	0.66	0.71	0.97	0.67	0.98	0.96
Generalized anxiety disorder	32	0.58	0.63	0.94	0.67	0.93	0.90
Post-traumatic stress disorder	19	0.70	0.95	0.93	0.60	0.99	0.94
Any anxiety disorder	61	0.72	0.89	0.86	0.76	0.94	0.88
Any disorder	78	0.72	0.86	0.86	0.81	0.90	0.87

The measures of agreement for the WB-DAT as compared with the SCID-I/P criterion for the DSM-IV Axis I disorders assessed are shown in Table 2. The Cohen's kappa measure of agreement ranged from acceptable to good (0.57-0.70) for MDD, PD+/-Ag, social phobia/social anxiety disorder, OCD, GAD, and PTSD. With the exception of GAD, the sensitivity (0.71-0.95) and specificity (0.87-0.97) for the major diagnostic categories assessed by the Web-Based Depression and Anxiety Test ranged were good. The sensitivity for GAD was somewhat lower (0.63) but acceptable. Positive predictive values were good (0.60-0.75) for MDD, OCD, GAD, and PTSD, and acceptable for PD+/-Ag and social phobia/social anxiety disorder.

## Discussion

These are preliminary data from a sample of subjects drawn from 2 studies of the treatment of MDD and from a community study of social and problem gamblers. Thus, the results of this study should be interpreted with some caution. However, these preliminary data suggest that the WB-DAT was reasonably accurate in identifying patients who met SCID-I/P criteria for MDD, SP, OCD, and PTSD. The WB-DAT was somewhat less accurate in identifying subjects with GAD, although this is likely due to the small sample size and the considerable comorbidity between MDD and GAD, as 35.41% of subjects who met SCID-I/P criteria for MDD also met SCID-I/P criteria for GAD.

Given the relatively small sample size in this study it is important to note that the Cohen's kappa, sensitivity, and specificity measures for the diagnoses of "any anxiety disorder" and "any disorder" were excellent. Thus, given that the true purpose of the WB-DAT is to produce output that can help initiate and encourage a discussion of symptoms and concerns between patients and health care providers in primary care, it appears to have the potential to be a useful tool in primary care.

In summary, the WB-DAT appears to do a reasonably good job of identifying people with MDD and/or an anxiety disorder. However, the results of this study require support from a larger validation study in primary care. The use of Web-based technology allows for constant improvements in screening modules and diagnostic algorithms in response to feedback from the results of validation studies. With the potential for continued development and validation, the WB-DAT provides a unique opportunity to make an important contribution to increasing recognition of MDD and the anxiety disorders in primary care.

## Abbreviations

AG: Agoraphobia
DSM-IV: Diagnostic and Statistical Manual of Mental Disorders, fourth edition
GAD: Generalized Anxiety Disorder
ICD-10: The International Classification of Diseases and Related Health Problems, tenth edition
MDD: Major Depressive Disorder
MINI: Mini-International Neuropsychiatric Interview
OCD: Obsessive-Compulsive Disorder
PD: Panic Disorder
PD+/-AG: Panic Disorder With and Without Agoraphobia
PTSD: Post-Traumatic Stress Disorder
SCID-I/P: Structured Clinical Interview for DSM-IV Axis I Disorders (Version 2.0/Patient Form)
SP: Social Phobia/Social Anxiety Disorder
WB-DAT: Web-Based Depression and Anxiety Test
